# Recreational Diving Impacts on Coral Reefs and the Adoption of Environmentally Responsible Practices within the SCUBA Diving Industry

**DOI:** 10.1007/s00267-016-0696-0

**Published:** 2016-04-07

**Authors:** Ronan C. Roche, Chloe V. Harvey, James J. Harvey, Alan P. Kavanagh, Meaghan McDonald, Vivienne R. Stein-Rostaing, John R. Turner

**Affiliations:** School of Ocean Science, Bangor University, Menai Bridge, Anglesey LL59 5AB UK; The Reef-World Foundation, Bwthyn Banadlen, Brynteg, Anglesey LL78 7JH UK

**Keywords:** Coral reef, Diving, SCUBA diving impacts, Tourism, Responsible diving

## Abstract

Recreational diving on coral reefs is an activity that has experienced rapidly growing levels of popularity and participation. Despite providing economic activity for many developing coastal communities, the potential role of dive impacts in contributing to coral reef damage is a concern at heavily dived locations. Management measures to address this issue increasingly include the introduction of programmes designed to encourage environmentally responsible practices within the dive industry. We examined diver behaviour at several important coral reef dive locations within the Philippines and assessed how diver characteristics and dive operator compliance with an environmentally responsible diving programme, known as the Green Fins approach, affected reef contacts. The role of dive supervision was assessed by recording dive guide interventions underwater, and how this was affected by dive group size. Of the 100 recreational divers followed, 88 % made contact with the reef at least once per dive, with a mean (±SE) contact rate of 0.12 ± 0.01 per min. We found evidence that the ability of dive guides to intervene and correct diver behaviour in the event of a reef contact decreases with larger diver group sizes. Divers from operators with high levels of compliance with the Green Fins programme exhibited significantly lower reef contact rates than those from dive operators with low levels of compliance. The successful implementation of environmentally responsible diving programmes, which focus on influencing dive industry operations, can contribute to the management of human impacts on coral reefs.

## Introduction

Coral reefs are a threatened, but globally important ecosystem, providing key services to local communities such as coastal defence, sediment production, and fisheries benefits (Bellwood et al. 2004; Moberg and Folke 1999; Rogers et al. 2015). In addition, they are a focus of global tourism, with the resulting economic activity generating a major portion of local income and providing a key source of livelihood in many coastal communities (Cinner 2014). Over recent decades, tourism activities benefiting from the pleasing aesthetics and biodiversity of coral reefs, primarily SCUBA diving and snorkelling, have experienced rapidly increasing numbers of participants globally (Barker and Roberts 2004; Davenport and Davenport 2006). Whilst initially considered to be ecologically benign, a cumulating body of research highlights a wide range of SCUBA diving impacts at frequently dived locations (Hawkins et al. 1999; Lamb et al. 2014; Tratalos and Austin 2001; Zakai and Chadwick-Furman 2002).

Damage to corals on dived reefs often occurs as a result of skeletal breakage, particularly in branching species (Guzner et al. 2010; Hasler and Ott 2008). Tissue abrasion can also result from diver contact (Hawkins et al. 1999), and a recent study reported a higher incidence of coral disease in areas heavily used for recreational diving (Lamb et al. 2014). In some instances, hard coral cover on reefs subjected to intensive SCUBA diving is lower than that on reefs less frequently dived (Hasler and Ott 2008; Hawkins and Roberts 1992; Tratalos and Austin 2001). Furthermore, diving-related activities may significantly impact a coral reef’s ability to withstand more widespread reef stressors such as climate change and coral bleaching events (Carilli et al. 2010; Marshall and Schuttenberg 2006). Due to the difficulties of effectively addressing global stressors, an emerging recommendation is the focus of coral reef management on local scales (e.g. Anthony et al. 2014). A frequent challenge facing managers and policy makers at local levels relates to the maximisation of tourism benefits whilst simultaneously reducing its environmental impacts (Roman et al. 2007).

The methodologies which have been developed to minimise the environmental impact of SCUBA diving on coral reefs can be summarised as follows: (1) managing or restricting diver numbers, (2) regulating the locations in which SCUBA diving activities occur, (3) regulating the types of equipment used, and 4) implementing programmes which seek to manage the methods used by the dive industry in providing their services. Restricting diver numbers is based on the concept of a reef dive site’s ‘carrying capacity’; a level beyond which diving impacts become readily apparent. This has been reported to vary between 5000 and 6000 dives per year (Hawkins et al. 1999) to up to 7000 dives per year (Schleyer and Tomalin 2000). Regulation of the areas in which SCUBA diving activities occur has been primarily implemented through the creation of underwater diving trails which aim to concentrate diving impacts within specific locations (e.g. Rios-Jara et al. 2013; Rouphael and Inglis 2002). Restriction of SCUBA diving equipment has focused on banning the use of accessories believed to increase reef contacts within marine protected areas such as gloves, muck sticks, or underwater cameras; however, such regulations are often unpopular within the SCUBA diving community (Poonian et al. 2010).

In comparison to restricting diver numbers, use of specific dive equipment or dive locations, improved management of the diving process by instructors and guides is infrequently cited as a method for reducing SCUBA diving impacts on reefs (Hasler and Ott 2008; Sorice et al. 2007). Nonetheless, levels of dive supervision underwater would intuitively appear to be linked to rates of reef contact, and when examined, the willingness of dive guides to intervene in correcting diver behaviour underwater has been found to significantly reduce diver contact rates (Barker and Roberts 2004).

One mechanism for potentially reducing diver impacts on reefs is the use of a pre-dive briefing to provide information on topics such as responsible diver behaviour, relevant regulations, and the environmental value of a dive site. Studies examining the effects of pre-dive briefings on diver impacts have produced varied results. Both Camp and Fraser (2012) and Krieger and Chadwick (2013) found that the inclusion of a pre-dive briefing reduced divers’ reef contact rates in the Florida Keys, similar to earlier research in the Red Sea (Medio et al. 1997). In contrast, Barker and Roberts (2004) found no effect of the inclusion within dive briefings of a request to refrain from touching the reef on divers’ reef contact rates around the Caribbean island of St. Lucia. It is possible that other diver characteristics such as qualification level or dive experience may affect the ability to respond to dive briefings, although several studies have failed to find a correlation between divers’ reef contact rates and experience (Camp and Fraser 2012; Chung et al. 2013; Luna and Pérez 2009). Alternatively, previous experience and possible affinity and attachment to a specific dive site may influence how closely divers follow pre-dive briefings and affect their behaviour underwater, as suggested by place attachment theory (e.g. Bricker and Kerstetter 2000; Halpenny 2010).

In addition to the utilisation of pre-dive briefings, environmentally responsible diving programmes employ a range of educational (e.g. coral identification workshops) and procedural tools (e.g. use of dive boat moorings, dive guide interventions underwater) to address diving impacts, and have been incorporated into tourism management strategies at many coral reef locations with high visitor numbers. Established programmes range from those with a primarily educational focus such as PADI AWARE, Blue the Dive in the United States, and REEF survey courses, to regional programmes with a policy background such as the NOAA Blue Star charter within the Florida Keys (Camp and Fraser 2012; Krieger and Chadwick 2013), and the Green Fins programme initiated by UNEP within South-East Asia (Hunt et al. 2013). Governments and reef managers seek evidence that the effort expended in implementing programmes translates into measurable benefits; however, research into the effectiveness of such programmes at influencing diver behaviour and reducing diving impacts is limited.

In this study, we focused on dive operators participating in the Green Fins diving programme at three major dive locations within the Philippines. The effects of dive operator compliance with the Green Fins programme on divers’ reef contact rates were studied, specifically examining the influence of diver supervision levels and dive guide intervention underwater. We also examined the influence of diver characteristics on the frequency of reef contacts made during dives.

## Methods

### Underwater Data Collection

**Fig. 1:**
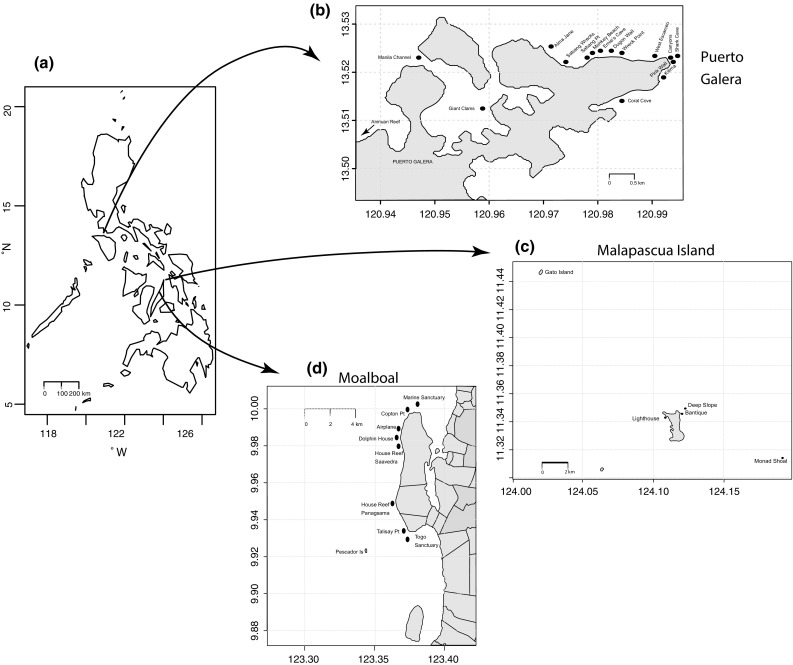
Map of the study locations within **a** the Philippines (*n* = 30), **b** Puerto Galera (*n* = 16), **c** Malapascua Island (*n* = 5), and **d** Moalboal (*n* = 9). *Dark points* indicate dive site locations visited

Research was carried out at three major dive locations within the Philippines with high numbers of Green Fins dive operators: Malapascua Island, Moalboal, and Puerto Galera. A total of 30 dive sites in this region were visited during the study: 16 dive sites in Puerto Galera, nine dive sites in Moalboal, and five dive sites around Malapascua Island (Fig. 1). Only patch (isolated) and fringing (running along the shoreline) reefs of similar topography were included, to minimise any potential effects of topography on diver behaviour and reef damage (Rouphael and Inglis 1998). Data were collected during several periods, starting during December 2012, continuing from May 2013 to June 2013, December 2013 to January 2014, and from March 2014 to April 2014.

### Dive Operator Compliance with the Green Fins Programme

Dive guides and guest divers from 44 dive operators participating in the Green Fins programme were followed during the underwater portion of the assessment for compliance with Green Fins environmental standards. Qualified Green Fins assessors accompanied divers and dive guides during normal diving excursions at each dive site, and followed randomly selected individuals from the group of divers entering the water on that day (method as Krieger and Chadwick 2013). Therefore, divers may have been aware that a Green Fins compliance assessment was taking place, but they were unaware that diver contacts with the reef were being specifically recorded. Green Fins environmental assessments and diver observations were conducted simultaneously. A detailed explanation of the Green Fins assessment methodology has been published previously (Hunt et al. 2013). In brief, the assessors evaluated regular diving business practices against a set of 15 code of conduct points, which range from providing coral and fish ID books in dive shops, to giving information on local marine protected areas and environmental regulations, and promotion of a “no-touch” diving policy. Each code of conduct point has an associated weighted score based on its potential threat to marine biodiversity. Following the assessment, the dive operator management was assigned a Green Fins total score, which benchmarks their level of compliance with the Green Fins standards.

### Diver Reef Contact

Divers were assigned a unique diver number, and then followed and observed underwater for the entire duration of their dive. The dive buddy pair followed was selected underwater, in an essentially random process, as the last pair of the sequence of divers behind the dive guide underwater. If the overall group was very large such that the dive guide could not be seen from the rear of the group, the pair immediately behind the dive guide was selected. A contact was recorded when any part of the diver’s body or equipment made contact with the reef or substrate during the dive. The part of the body or item of equipment making contact with the reef was recorded as follows: hand, fin, knee, camera, muck stick (a handheld stainless steel or aluminium rod approximately 30 cm in length) and equipment (e.g. tank, submersible pressure gauges, octopus regulator), and multiple (parts of the body and equipment simultaneously). The time during the dive at which the contact occurred was also recorded. The type of substrate contacted was recorded according to the following categories: live hard coral, dead hard coral, live soft coral, rubble, sand, reef framework, and other marine life. If observable damage (i.e. breakage, obvious physical damage, or injury) occurred as a result of the contact this was recorded, together with the apparent awareness of the diver to the contact, regardless of damage caused.

The number of divers per dive guide (who had a qualification level of either dive instructor or dive master) and the number and timing of any interventions made were recorded. Interventions were defined as an event in which the dive guide intervened in diver behaviour through signalling or demonstrating correct behaviour in order to minimise or prevent contact with the reef.

### Diver Characteristics Survey

Following dive completion, divers that had been observed underwater were asked to complete a survey to determine diver characteristics. A 100 % response rate was achieved for this brief survey. The survey comprised questions on demographic characteristics (gender and nationality), diver qualification level, total number of lifetime dives, and number of dives previously completed at the dive site visited that day. Data relating to the use of a camera on the dive and the type of camera (classes of non-specialist point-and-shoot systems or single lens reflux (SLR) cameras in specialist housings (Inglis and Rouphael 2001) were recorded. Divers were also asked to rate their perception of the ecological condition of the dive site and their enjoyment of the dive according to a Likert-type scale.

### Statistical Analyses

Diver characteristics with potential to influence underwater behaviour were categorised as the following factors: diver qualification level (three levels), dive experience (five levels), and previous number of dives at site (three levels). Dive supervision was analysed by defining dives accordingly: those where the number of divers per dive guide was low (<3) versus those that were high (>3). Based on recorded dive times, contacts and interventions were allocated to either the start (1st third of individual dive time), mid (2nd third of individual dive time), or end (final third of individual dive time) phases of dives. Compliance with the Green Fins approach was determined by utilising diver contact rates and dive guide intervention rates as at the dependent variables of interest, and by defining dive operators according to those who had received a high score (above the median score) versus those with a low score (below the median score) on the most recent conducted Green Fins assessment (for details see Hunt et al. 2013). The Shapiro–Wilk test was used to test diver contact rates for normality and data were square-root transformed to satisfy assumptions required for parametric testing (Sokal and Rohlf 1995). Prior to performing ANOVA, the Fligner–Killeen test was utilised to test variables for homogeneity of variance (Conover et al. 1981). Statistical analysis was conducted using 95 % confidence limits and carried out using the R program (R Development Core Team 2014).

## Results

### Diver Characteristics

A total of 100 SCUBA divers were observed at three diving locations within the Philippines (Table 1). The majority (72 %) of these divers were male, and diving experience ranged from those completing diving training to those who were instructors elsewhere with experience of hundreds of dives. Overall, experience levels were high: 50 % of the divers in this study had completed 100 or more dives, 11 % from 50 to 100 dives, 27 % from 50 to 10 dives, and 12 % had completed <10 dives. Of these divers, the majority (88 %) made contact with the reef at some point during the observed dives. Camera systems were carried by 55 % of divers; camera-wielding divers accounted for 52.7 % of the total contacts made with the reef. Of divers who utilised a camera, 35 % carried a non-specialist compact type and 20 % carried an SLR type within a specialist underwater housing. A small proportion (8 %) of divers were observed to utilise a muck stick during dives. Post-dive enjoyment rating was significantly correlated (*r* = 0.56, *P* ≤ 0.001) with the diver’s assessment of the dive site’s ecological health.

**Table 1:** Summary of diver characteristics

Dive certification agency	
Association of Diving School International (ADS)	1
Professional Association of Diving Instructors (PADI)	67
National Association of Underwater Instructors (NAUI)	6
Scuba Schools International (SSI)	5
British Sub Aqua Club (BSAC)	3
Confédération Mondiale des Activités Subaquatiques (CMAS)	3
Other/not given	15
Qualification level (or equivalent)	
Open water	26
Advanced open water	33
Rescue diver	8
Dive master	12
Instructor	10
Other	11
Previous times dived at site	
0	10
1	28
2–5	18
5–10	4
10+	11
Not given	29
Lifetime diving experience	
0–5	6
6–10	6
11–25	16
26–50	11
51–100	11
101+	50

### Diver Behaviour Underwater

**Fig. 2:**
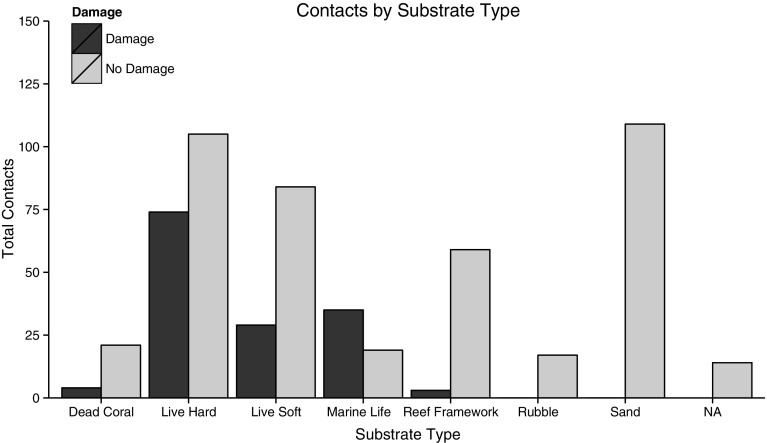
Recreational diver reef contacts by substrate type. *Dark bars* indicate damaging contacts, and *lighter bars* indicate no observable damage

Mean (±SE) dive time was 49.3 ± 0.42 min. A total of 573 diver contacts with the reef were recorded during all assessed dives. The mean (±SE) number of reef contacts made per diver over the course of the dive was 5.7 ± 0.67. The mean (±SE) diver contact rate per dive was 0.12 ± 0.01 contacts per minute and the median contact rate was 0.07 contacts per minute. Of the contacts recorded, 25.3 % (*n* = 145) resulted in observable damage to the reef or reef marine life. Of the 179 contacts that occurred with live coral, 41.3 % (*n* = 74) resulted in observable damage. For contacts made with soft coral, 25.7 % (*n* = 29) resulted in damage (Fig. 2) and for all other reef marine life, 64.8 % (*n* = 35) of contacts resulted in observable damage.

**Fig. 3:**
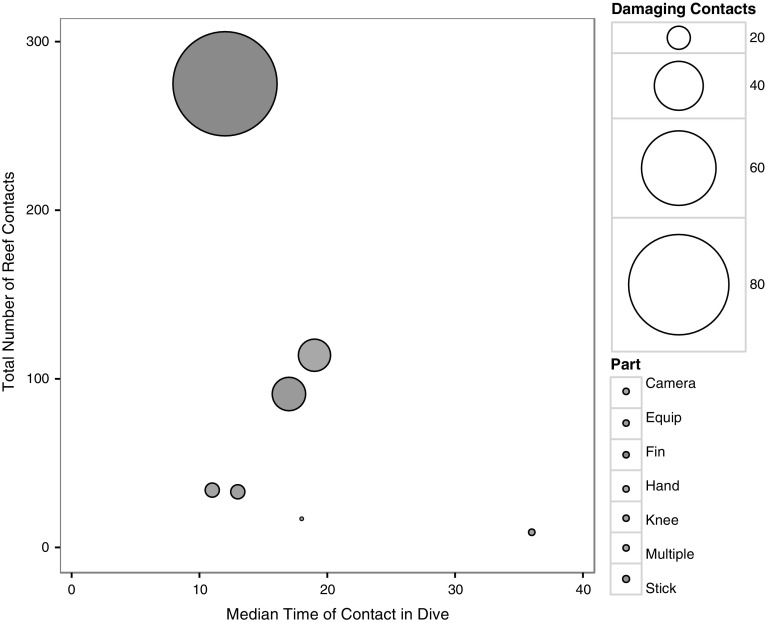
Recreational diver reef contacts by the item of equipment or part of body which made contact. The *x*-axis is the median time in dive at which those contacts occurred, to indicate the central tendency within dive time for contacts with that item of equipment to occur. Total number of contacts is graphed on the *y*-axis and circle size corresponds to the number of damaging contacts

Most contacts were made with fins (45.5 %, *n* = 261); however, hands (19.5 %, *n* = 112) and dive equipment (15.9 %, *n* = 91) were also major contributors to the total number of contacts made with the reef (Fig. 3). Contacts made with a camera (77.7 %) accounted for the highest proportion of contacts which resulted in damage, followed by contacts made with the knee (43.3 %), multiple body and equipment parts (38.2 %), equipment (30.7 %), fins (29.8 %), hands (24.7 %), and muck sticks (23.5 %). For the majority of contact events (63.4 %, *n* = 366), divers were recorded as being aware of the contact they had made during the dive.

**Fig. 4:**
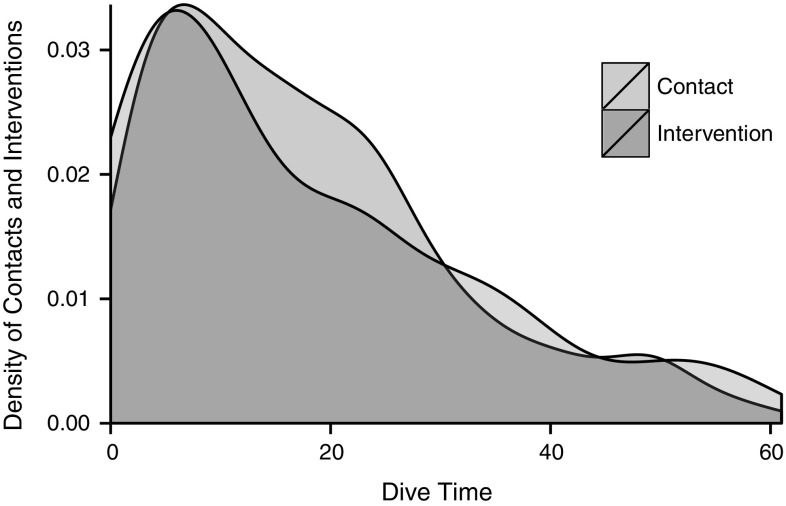
Kernel density plot (smoothed histogram) illustrating the distribution of all recorded diver reef contacts and dive guide interventions at the time in the dive at which they took place

A total of 81 interventions were observed (in comparison to 573 reef contacts—see Fig. 4 for the distribution of contacts and interventions); interventions occurred on 37 % of dives and the mean (±SE) intervention rate was 0.04 ± 0.003 interventions per min. The majority of interventions (80.2 %) took place in the absence of a contact (e.g. buoyancy correction) or prevented contact with the reef before it occurred. The contact rate in early portions of dives was 0.208 ± 0.02 contacts per min, in mid-portions 0.144 ± 0.01, and in the later portions 0.06 ± 0.01. This variation in contact rate was statistically significant (ANOVA, *f* = 6.922, *P* ≤ 0.001). Likewise, the intervention rate was higher in the early portion of dives (0.07 ± 0.02 per min), than the mid- (0.04 ± 0.01 per min), and late portions (0.04 ± 0.01 per min), and this variation in intervention rate was statistically significant (ANOVA, *f* = 3.317, *P* ≤ 0.04).

There was no relationship between reef contact rate per minute and the qualification level of divers (ANOVA, *f* = 0.27, *P* = 0.6) or lifetime numbers of dives completed (ANOVA, *f* = 0.003, *P* = 0.9). There was also no relationship between the number of times divers had previously dived the dive site and the contact rate per minute during the dive (ANOVA, *f* = 1.516, *P* = 0.222). The mean (±SE) contact rate of divers who carried a camera was 0.12 ± 0.02 contacts per min, which was equal to divers who did not carry a camera (0.12 ± 0.02 contacts per min). The mean (±SE) contact rate of divers who carried a muck stick was 0.22 ± 0.06 contacts per min, significantly higher (*t* test, *P* = 0.03) than divers who did not carry a muck stick (0.11 ± 0.01).

### Effects of Supervision and Dive Operator Characteristics on Diver Behaviour

The mean (±SE) contact rate of divers when the ratio of divers to dive guides was high was 0.12 ± 0.02 contacts per min. When the diver-to-dive guide ratio was low, the mean (±SE) contact rate was 0.14 ± 0.02 contacts per min. This difference was not statistically significant (ANOVA, *f* = 0.896, *P* = 0.35). The mean (±SE) frequency of interventions when the ratio of divers to dive guides was high was 0.01 ± 0.003 per min. When the diver-to-dive guide ratio was low, the mean (±SE) frequency of interventions was 0.03 ± 0.007 contacts per min. The difference in the frequency of interventions was statistically significant (ANOVA, *f* = 4.81, *P* = 0.03).

**Fig. 5:**
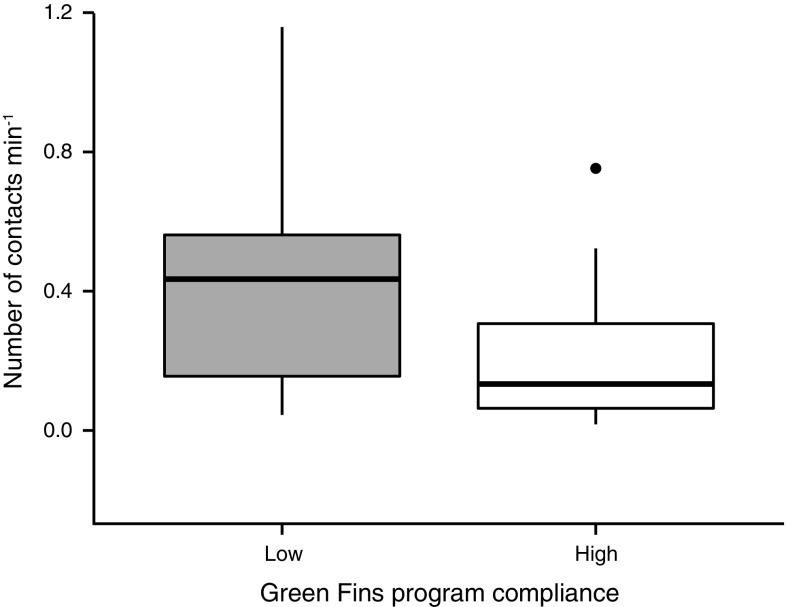
Comparison of the rates of reef contacts made by recreational SCUBA divers from low Green Fins compliance diver operators versus high Green Fins compliance dive operators

The mean (±SE) intervention rate was 0.05 ± 0.02 per min for dive operators with low Green Fins compliance and 0.04 ± 0.01 per min for dive operators with high Green Fins compliance. This difference was not statistically significant (ANOVA, *f* = 0.396, *P* = 0.532). The mean (±SE) number of contacts per minute for dive operators with low Green Fins compliance was 0.40 ± 0.07, whilst for dive operators with high Green Fins compliance, mean contact rates were 0.19 ± 0.03 per min. This difference was statistically significant (ANOVA, *f* = 9.278, *P* = 0.004, Fig. 5).

## Discussion

Identifying factors and policy measures which influence SCUBA diver behaviour underwater can help coral reef managers determine where to most effectively focus effort and funding with respect to dive management. In this study, we found that 88 % of the divers observed made at least one contact with the reef at some point during their dive, although a significant portion (36 %) appeared unaware of the contact they made with the reef. This finding is similar to previous studies which have reported overall levels of contact amongst divers ranging from 71 % (Krieger and Chadwick 2013) to 97 % (Camp and Fraser 2012; Luna and Pérez 2009). In addition to overall contact levels, some studies have also quantified reef contacts either as the mean number of contacts per diver over the duration of a dive or the diver contact rate per minute of dive time. The mean contact rates of 5.7 contacts per dive, or 0.12 contacts per min, which we observed at dive sites in the Philippines are lower than those previously reported. For instance, Chung et al. (2013) recorded a mean of 14.7 contacts per dive amongst (predominantly low experience) divers in Hong Kong, whilst Krieger and Chadwick (2013) reported a mean contact rate of 0.31 contacts per min in the Florida Keys, and Rouphael and Inglis (1998) recorded 0.54 contacts per min at dive sites within the Australian Great Barrier Reef.

All divers observed within the present study were diving with operators participating to various degrees in the Green Fins environmentally responsible diving programme. Two previous studies examining the effect of participation in an environmentally responsible diving certification programme in the Florida Keys observed lower contact rates among divers from participating diver operators. Camp and Fraser (2012) recorded a lower contact rate of 0.16 contacts per min for divers from dive centres who participated in the Blue Star charter programme, compared with 0.37 contacts per min for non-Blue Star dive centres. In a later study, Krieger and Chadwick (2013) also reported a difference of 0.37 contacts per min versus 0.25 contacts per min when dive operators were participants in the Blue Star programme. There are several potential reasons for the observed decrease in diver contact rates in these studies, which are also relevant to our findings. Divers who are more conservation aware and who contact the reef less may preferentially choose to dive with environmentally ‘accredited’ dive operators; indeed, this assumption partially drives dive operator participation in such programmes. In the present study, this effect would be minimised as all dive operators were participating in the same environmentally responsible dive programme.

Underwater interventions by dive guides have been suggested to be the most successful deterrent to diver contact with reefs (Barker and Roberts 2004). In this study, there was no significant difference in the intervention rates between dive centres of high and low Green Fins compliance. Therefore, we cannot attribute the observed difference in diver reef contact rates to differences in intervention rates between these two groups. However, we did find that levels of diver supervision influenced intervention rates in a logical fashion, with higher intervention rates associated with higher levels of diver supervision. Whilst previous studies have recommended that high levels of diver supervision underwater would be beneficial in facilitating interventions (Barker and Roberts 2004; Krieger and Chadwick 2013), in this study we have empirically demonstrated the existence of this relationship.

Additionally, the administration of a pre-dive briefing can influence diver contact rates underwater (Medio et al. 1997). The Green Fins programme incorporates the use of a pre-dive briefing that emphasises the importance of refraining from contacting the reef, which would be expected to result in lower diver contact rates. In addition to the presence or absence of a dive briefing, there is evidence that the quality and content of the briefing influences contact rates (Camp and Fraser 2012). We therefore suggest that differences in contact rates between high and low Green Fins compliance dive operators observed in the present study may be partially due to variation in the quality of dive briefings, and dive briefing may be more effective in smaller groups. Additional factors may also influence the observed difference in contact rates between high and low Green Fins compliance dive operators, in particular the attitude and sincerity of dive operators towards marine conservation, the information provided in dive centres, and leading dives by positive example underwater (Camp and Fraser 2012). The specific factors (e.g. dive briefing, group size, environmental information provision) which relate to the observed differences in diver reef contact rates between high and low Green Fins compliance operators are the subject of on-going research.

When examining the part of the body or dive equipment which made contact with the reef, we found that the majority of contacts were made with fins, in agreement with Krieger and Chadwick (2013) and Rouphael and Inglis (1998). These contacts occurred most frequently during the early portion of the dive, between 10 and 15 min (Fig. 4), and are therefore likely to reflect adjustment of buoyancy occurring before the main portion of the dive, as suggested by previous studies (e.g. Camp and Fraser 2012). Given the high experience levels of the divers observed in our study, it is perhaps surprising that issues with buoyancy control remain. Regardless, this finding supports management measures which seek to restrict SCUBA diving entry points to specific areas of a reef (e.g. Krieger and Chadwick 2013; Meyer and Holland 2008). Diver experience levels would intuitively be expected to influence the number of reef contacts underwater; however, we did not find a relationship between experience or qualification level and the frequency of reef contacts made, in agreement with Chung et al. (2013) and Camp and Fraser (2012). These results, together with the finding that the majority of divers appeared to be aware of the contacts they were making with the reef, suggest that diver education and raising awareness across all experience levels could have a positive effect in reducing reef contacts.

Studies examining the effect of carrying camera equipment on the frequency of diver contacts with the reef have produced conflicting results. Whilst Rouphael and Inglis (2002) and Uyarra and Côté (2007) found that camera equipment increased the chance of interacting with the reef, others have not reported an effect of carrying camera equipment (Camp and Fraser 2012). In our study, reef contact rates of divers carrying a camera were equal to those not carrying any camera equipment. We note that the proportion of divers carrying a camera system in the present study (51 %) was higher than that recorded by previous studies; Camp and Fraser (2012) found that 14.1 % of divers in the Florida Keys carried camera equipment, while Krieger and Chadwick (2013) reported that 20.4 % of divers carried an underwater camera. The high levels of underwater camera usage observed in this study may be related to high diver experience levels, but are also likely a result of the increase in the availability and affordability of compact varieties of underwater camera.

The use of a muck stick (a handheld stainless steel or aluminium rod approximately 30 cm in length) as a means for a diver to stabilise themselves, whilst underwater is a controversial practice within the SCUBA diving industry (Cooper 2011), and one which has been banned in the Red Sea (CDWS 2010). A concern amongst representatives of the diving industry is the use of muck sticks to manipulate animals unnecessarily—pushing animals out of holes for better viewing, stressing animals to show customers their stress behaviour (e.g. an octopus changing colour), and physically breaking hard coral to be used in photographs. Proponents of their usage suggest that they may help prevent reef contact, or reduce the level of damaging contact. Our data found that divers carrying a muck stick contacted the reef more than those who did not, but muck sticks caused the lowest proportion of obviously damaging contacts of body and equipment parts which were observed to contact the reef. However, as this study was not designed specifically to examine the use of muck sticks, and the number of divers who carried a muck stick was small, we suggest that additional research is needed to more robustly determine their impacts.

It has previously been noted that dive guides customarily perform different roles at dive locations globally; at some locations, they act primarily to lead the dive group around the reef, whilst at others, pairing with and closely supervising individual divers throughout the course of a dive (Krieger and Chadwick 2013). We found that dive guide interventions followed a similar temporal pattern to reef contacts during the dive; these were highest in the initial stage of the dive, and decreased towards the end of the dive (Fig. 4). It is revealing that interventions do not remain constant during the dive; this suggests that dive guides carry out the closest supervision during the initial phase of the dive and then switch to a ‘dive leader’ role at the front of a dive group. At dive sites within the Philippines, encouraging dive guides to continue interventions when reef contacts occur and demonstrating correct behaviour throughout the entire course of a dive could result in further reductions in divers’ reef contacts.

## Conclusion

This study provides evidence that the effective implementation of environmentally responsible practices, via programmes designed to reduce diving impacts, may translate to reduced diver reef contacts. The finding of low overall diver reef contact rates in the present study, comparable to other locations worldwide where environmentally responsible dive programmes have been implemented, provides additional support for the effectiveness of the Green Fins approach. Differences observed between high and low Green Fins compliance dive operators indicate that levels of engagement within a dive impact minimisation programme can influence the number of reef contacts made by divers.

Many diver characteristics which might intuitively be expected to impact reef contact rates, such as level of qualification and overall experience, were not significant influencing factors in this study, and high versus low levels of Green Fins compliance did not influence the number of interventions made by dive guides underwater. We suggest that dive operator’s behaviours and attitudes towards conservation measures are more important factors in influencing diver reef contact rates. For continued economic benefit and conservation of Philippine reef dive locations, we recommend that management measures facilitate high levels of compliance with environmentally responsible diving programmes to reduce the impact of diving on coral reefs. High levels of diver supervision to aid dive guide intervention in the event of reef contacts and the concentration of dive entry points to specific reef locations should also be emphasised within environmentally responsible dive programmes.
